# A Self-Powered Strain Sensor Applied to Real-Time Monitoring for Movable Structures

**DOI:** 10.3390/s22166084

**Published:** 2022-08-15

**Authors:** Yan-Kuei Wu, Sheng-Chih Shen, Chun-Yen Lee, Yen-Ju Chen

**Affiliations:** Department of Systems and Naval Mechatronic Engineering, National Cheng Kung University, Tainan 70101, Taiwan

**Keywords:** polyvinylidene difluoride, strain sensor, near-field electrospinning

## Abstract

This study uses near-field electrospinning (NFES) technology to make a novel self-powered strain sensor and applies it to the real-time monitoring of a bending structure, so that the measurement equipment can be reduced in volume. A self-powered strain sensor consists of polyvinylidene difluoride (PVDF) fibers, a PDMS fixed substrate, and an aluminum electrode. PVDF fibers are spun with DMSO and acetone using NFES technology, with a diameter of about 8 μm, Young’s modulus of 1.1 GPa, and piezoelectric effect of up to 230 mV. The fixed substrate is a film made of PDMS by thermal curing, then adhered to the PDMS film surface of the sheet Al metal as an Al electrode, and then combined with PVDF fiber film, to become a self-powered strain sensor. As a result, the XRD β value of the self-powered strain sensor reaches 2112 and the sensitivity is increased by 20% over a traditional strain sensor. The cumulative angle algorithm can be applied to measure the angular change of the object over a unit of time or the cumulative displacement of the object over the entire period of motion. The experimental results demonstrate that the self-powered strain sensor combined with the angle accumulation algorithm may be applied to monitor the bending structure, thereby achieving continuous measurements of bending structure changes, and improving on traditional piezoelectric sensors, which can only be sensed once. In the future, self-powered strain sensors will have the ability to continuously measure in real-time, enabling the use of piezoelectric sensors for long-term monitoring of structural techniques.

## 1. Introduction

Sensors convert applied physical quantities into electrical signals. In various conversion mechanisms, piezoresistive [1], capacitive [2], piezoelectric [3], and frictional [4] sensors all quantify external changes in electrical signals. Traditional strain sensors are often used to estimate displacement or force in mechanical systems. In recent years, matching industrial development needs, wearable, high-sensitivity, and high-stability micro-sensors [5] have become the hotspots of research on sensor networks, the Internet of Things (IOT), and miniaturized wearable electronics products. In motion sensing, portable micro-sensors are extremely important for real-time measurement of the acceleration, velocity, and force of objects, for monitoring the body’s motion parameters and health conditions in biomedical practice [6,7,8]. At present, most sensors require a battery device, and they cannot be separated from the battery device to work autonomously or self-powered [9].

Self-powered sensors convert mechanical kinetic energy into electrical signals using piezoresistive, piezoelectric, capacitive, and frictional methods. To achieve self-power, common sensors are made of polyvinylidene difluoride (PVDF) as the main material. As a piezoelectric molecular material [10], PVDF features good flexibility, a simple manufacturing process, high strain level, strong resistance to mechanical deformation, and low manufacturing cost. PVDF’s piezoelectric sensors, with their simple process, wide material selection, and low-cost advantages, are considered the best material choice for self-powered sensors. PVDF’s piezoelectric sensors are manufactured in a number of ways, such as spin-coating [11] and electrostatic spinning [12]. Among them, electrostatic spinning technology can make a micro-nanometer-level sensor [13], increasing the surface area ratio of the sensor and thus the effective contact area and the cumulative charge density.

High-sensitivity piezoelectric elements are manufactured, and the most commonly used process for this is electrostatic spinning. Cooley et al. [14] proposed a patent related to electrostatic spinning for a technology that can produce actuators [15] and sensors, with a process accuracy reaching nanometer grade in recent years. NFES, a manufacturing process, produces microfibers from solution by applying an electric field. When applying enough voltage to the solution, the droplet will be drawn out because the electric charges in the solution eliminate the surface tension. Thanks to the adhesion between molecules in the droplet, a flow then be formed. After the liquid in the flow evaporates over time, a fiber shows up on the collecting board. NFES is simple, controllable, and low cost. Furthermore, the fibers collected from NFES can even achieve a micron scale and may be self-powered. NFES technology can create high-precision ultra-fine nanowires and 3D, patterned, stacked printing [16]. Moreover, self-powered strain sensors developed using NFES technology can break through the current shortcomings of strain sensors requiring a power supply. NFES technology can write PVDF directly on flexible substrates, making self-powered nanometer devices, which can be applied to implantable biomedical devices [17], wireless sensors, and portable electronic devices [18].

A traditional strain sensor mainly changes the resistance of the sensing element by applying an external force, then determines the bending of the object according to the resistance value. The piezoelectric element is made by the NFES technology [19,20], and a voltage is generated when the piezoelectric element is affected by an external force [20]. Using these voltages, the sensor can become a self-powered strain sensor, and no additional battery device is required. The utilization of the space is greatly improved, and the object is miniaturized. In that context, this study develops a sensor using PVDF fiber without a battery device, i.e., a self-powered strain sensor, and designs a cumulative angle algorithm to improve traditional piezoelectric sensors that cannot be continuously judged. The idea came from [21]. The sensor can take continuous position measurements like a traditional strain sensor, but without resetting, and does not require a battery device like a traditional strain sensor does for its power supply. 

## 2. Design of Self-Powered Strain Sensor

**Figure 1:**
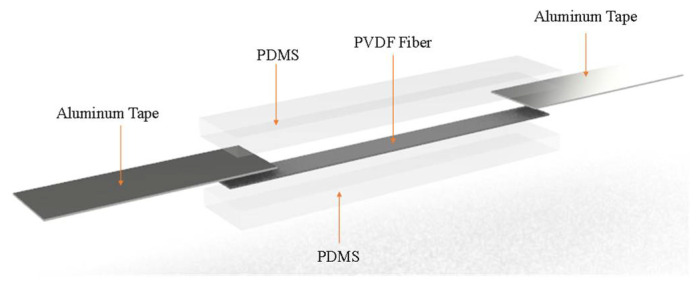
Structure of self-powered strain sensor.

In this paper, the sensor is mainly composed of PVDF and PDMS. In basic bending mechanics, Young’s modulus of PDMS film is 1.32~2.97 MPa, the PVDF fiber modulus of PVDF spun by the NFES process is about 1.1 GPa, and Young’s modulus of PDMS film is larger than that of PVDF fiber. The self-powered strain sensor is constructed as shown in Figure 1. The intermediate layer is a PVDF fiber film and the upper and lower layers are PDMS film material. A pair of aluminum sheets are attached to the PVDF fiber film to form aluminum electrodes. The aluminum electrodes will output the voltage signal generated by PVDF fiber film. Therefore, when the self-powered strain sensor is subjected to external forces, and this leads to bending, PVDF fibers can come under axial stress and produce strain to convert into electrical energy. Afterward, the voltage is generated when the PVDF fiber is affected by an external force. Using these voltages, the sensor can become a self-powered strain sensor, so no additional battery device is required.

The sensor in this study is composed of multiple layers of materials as a composite structure, and the displacement equation of the sensor is calculated by considering the elastic coefficient ratio *n* of each layer’s structure. The aluminum electrode layer and insulating adhesive are extremely thin compared to the other material layers, so the aluminum metal layer and insulating adhesive are not considered when analyzing the overall structural variation. When the positive force on the piezoelectric strain sensor has to be balanced,

(1) $$F=\int_{A}\rho dA=\int_{A}-\frac{Ey}{\rho }dA=0$$

*ρ* is a constant, and since it is composed of two materials, Young’s modulus is not a constant. Meanwhile, the neutral axis condition is

(2) $$\int_{A}E\left(y\right)ydA=0$$

Using Equation (2), the position of the neutral axis of the sensor can be calculated. Since the modulus of the PDMS film of the bottom layer is larger than that of the PVDF fiber film, the neutral axis is biased toward the bottom layer, and the PVDF fiber film is remote from the neutral axis. When the sensor substrate PDMS film Young’s modulus is *E*_1_, the thickness is *t*_1_, the PVDF fiber film Young’s modulus is *E*_2_, the thickness is *t*_2_, the width of the sensor is b, the neutral axis is located at the *δ* position below the two interfaces of the PDMS film and PVDF fiber film, the strain of the PVDF fiber film is

(3) $$\delta =\frac{nt_{1}{}^{2}-t_{2}{}^{2}}{2\left(nt_{1}+t_{2}\right)}$$

where $\frac{E_{2}}{E_{1}}=n$ is the elasticity coefficient.

(4) $$\varepsilon =-\frac{1}{\rho }\left(t_{2}+\delta \right)$$

The piezoelectric structure in this study must be stressed and strained to generate a voltage. This study uses the calculation of the neutral axis position to avoid the neutral axis being located at the position of the PVDF fiber layer, resulting in the reduction of the strain and the lowering of the PVDF fiber power-generation efficiency. Therefore, the PDMS film thickness *t*_1_ of this sensor is designed to be 1.7 mm. Young’s modulus *E*_1_ of the PDMS film is 2.15 MPa. The PVDF fiber thickness *t*_2_ is 3.81 μm. Young’s modulus *E*_2_ of the PVDF fiber is 1.2 GPa. The sensor structure width is 1.5 cm. The elasticity coefficient is 1.77. Finally, the material parameters and sensor geometry are brought into Equation (3), and the strain of the PVDF fiber can be obtained *ε*.

(5) $$\delta =\frac{1.7727\times {\left(1.7\times 10^{-3}\right)}^{2}-{\left(3.813\times 10^{-6}\right)}^{2}}{2\left(1.773\times 1.7\times 10^{-3}+3.813\times 10^{-6}\right)}=8.49\times 10^{-4}$$

(6) $$\varepsilon =-\frac{1}{\rho }\left(3.81\times 10^{-6}+8.49\times 10^{-4}\right)$$

Various parameters affecting the PVDF fiber film strain value difference in the structure of the sensing element can be determined by estimating the PVDF fiber film strain value through Equations (5) and (6). These include the ratio of the elastic coefficient of the PDMS film to the PVDF fiber film. The larger the elastic coefficient ratio, the higher the position of the PVDF fiber film from the neutral axis. With parameters such as a short structure length, it can help the structure to obtain a better strain value, and it can provide an ideal voltage signal for the conversion of Formula (7). It may also provide a reasonable design reference for the structural design and material selection of the self-powered strain sensor.

(7) $$V=d_{33}\times \delta \times L$$

where *L* is the length of the PVDF fiber, *δ* is the stress, and *d_33_* is the piezoelectric parameter.

## 3. Process of Self-Powered Strain Sensor

**Figure 2:**
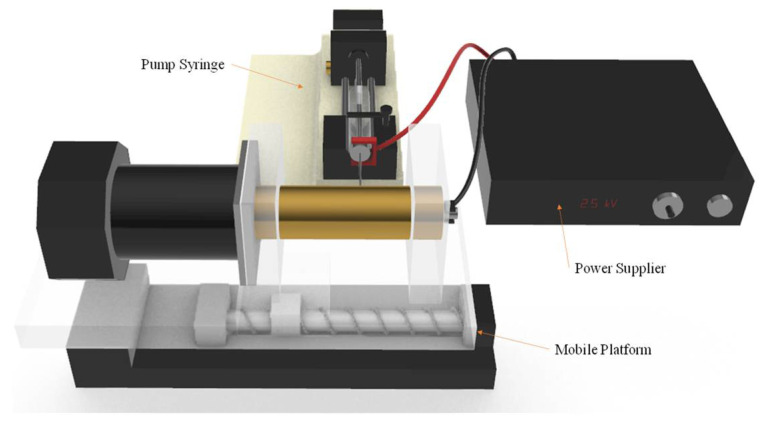
Schematic diagram of near-field electrospinning (NFES) equipment.

In this study, the self-assembled near-field electrospinning (NFES) equipment is as shown in Figure 2. This collects PVDF fiber using rotational technology to improve the traditional planar collection method, so that the electrospinning length is no longer limited. Making up the rotational technology, we use a copper circular cylinder with a DC motor as a collector. By setting the rotation speed (RPM) of the cylinder with the DC motor and shifting the speed of the platform on the *x*-axis, we can immediately obtain PVDF fiber in a good amount. When the platform moves back and forth, the collected PVDF fibers will overlap. This densely overlapped PVDF fiber looks like a thin film, so it is called PVDF fiber film in this paper. The equipment for the near-field electrospinning process includes a syringe, a precision flow control pump, a stainless steel needle holder, an *x*-axis movement platform, a high-voltage power supply, a DC motor, and a hollow tube collection device. The sensing element of the piezoelectric sensor is PVDF fiber film.

The PVDF fiber film is manufactured by drum near-field electrospinning. Before the sensing element is spun, the PVDF solution needs to be prepared. The PVDF powder, CH_3_COCH_3_, and dimethyl sulfoxide (DMSO) are mixed in a ratio of 1:3:2, and stirred for 1 h with a magnet stirrer. The PVDF mixed solution is bubbly after stirring, so it is allowed to stand for 1 h at room temperature with the aim of eliminating the bubbles; otherwise, they will affect the piezoelectric characteristic. The experimental design for drum near-field electrospinning is shown in Figure 2. The main equipment are the pump thruster, the power supply, and the collecting platform; the speed of the pump thruster is 160 μL/h using a 25 G needle tip (inner diameter 0.26 mm, needle length 46 mm), the voltage supply is 2.5 kV, the positive electrode of the voltage supply is fixed to the needle tip, and the negative electrode is fixed to the collecting plate. The NFES distance is less than 5 mm. NFES is easier to collect into neat PVDF fiber than FFES. The distance between the collecting platform and the needle tip depends on the humidity of the environment, the nature of the solution, the working voltage, etc. When there is a short distance, it is easy to generate an electrostatic reaction; when there is a long distance, is not easy to generate a Taylor cone and so we cannot generate PVDF fiber. The distance between the needle tip and the collecting platform in this experiment is 1 mm, the rotation speed of the copper cylinder is 1500 rpm, the spinning time is 1 h, and the environmental humidity is 25%.

**Figure 3:**
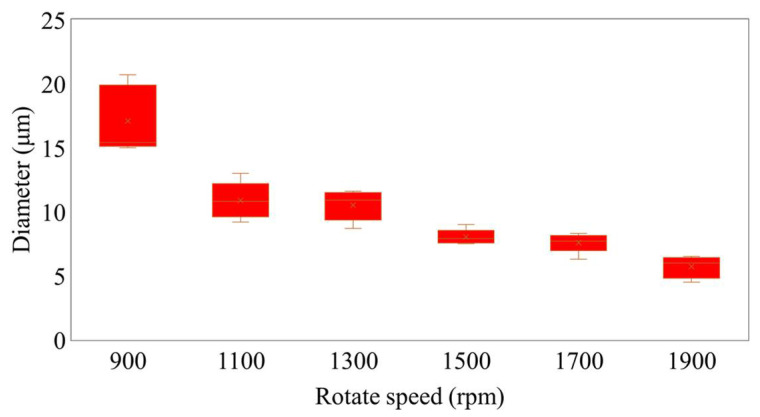
Relationship between the rotation speed of the hollow cylinder and the diameter of PVDF fibers.

**Figure 4:**
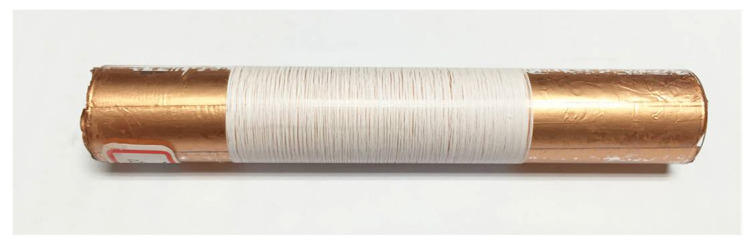
PVDF fibers collected by the hollow copper cylinder.

When we carried out this experiment, a near-field electrospinning process was used to fabricate PVDF piezoelectric fibers. Electrospun fibers are collected by a self-developed rotating collection device. By rotating the hollow cylinder with the *x*-axis moving platform, orderly and uninterrupted PVDF fibers could be collected. In this study, the relationship between the rotation speed of the drum and the PVDF fiber was investigated. The experimental parameters were fixed such as the supply voltage of 2.5 kV and the collection spacing of 1 mm. The speed of the *x*-axis moving platform was 5 mm/s, and only the rotation speed of the cylinder was changed. When other parameters are fixed, piezoelectric fibers with an exit diameter of 17 μm can be obtained when the cylinder speed is 900 rpm; piezoelectric fibers with a diameter of 12 μm can be collected when the cylinder speed is 1100 rpm; piezoelectric fibers of 9.5 μm can be obtained when the cylinder speed is 1300 rpm; piezoelectric fibers of 9.2 μm can be obtained at 1500 rpm; piezoelectric fibers of 7.3 μm can be obtained at 1700 rpm; piezoelectric fibers of 5.1 μm can be obtained at 1900 rpm. The fibers are sprayed and deposited on the cylinder. When the drum rotation speed is too slow, the collection speed will be slower than the injection speed. The piezoelectric fibers will be deposited on the cylinder too fast, accumulate, and cause deflection. When the rotating speed of the cylinder is greater than the spinning speed, the piezoelectric fibers deposited on the cylinder will be pulled by the cylinder, affecting the wire diameter of the piezoelectric fibers. When the speed is too fast, the electrospinning is easily pulled and broken. As a result, the collected electrospinning is discontinuous. Figure 3 shows the relationship between the rotation speed of the cylinder and the wire diameter for a fixed 2.5 kV voltage. Through the rotation of the cylinder and the movement of the platform, a large number of parallel piezoelectric fibers were quickly collected in this experiment. As shown in Figure 4, the fibers collected by the controlled rotating collection device were numerous and densely overlapped like a thin film.

**Figure 5:**
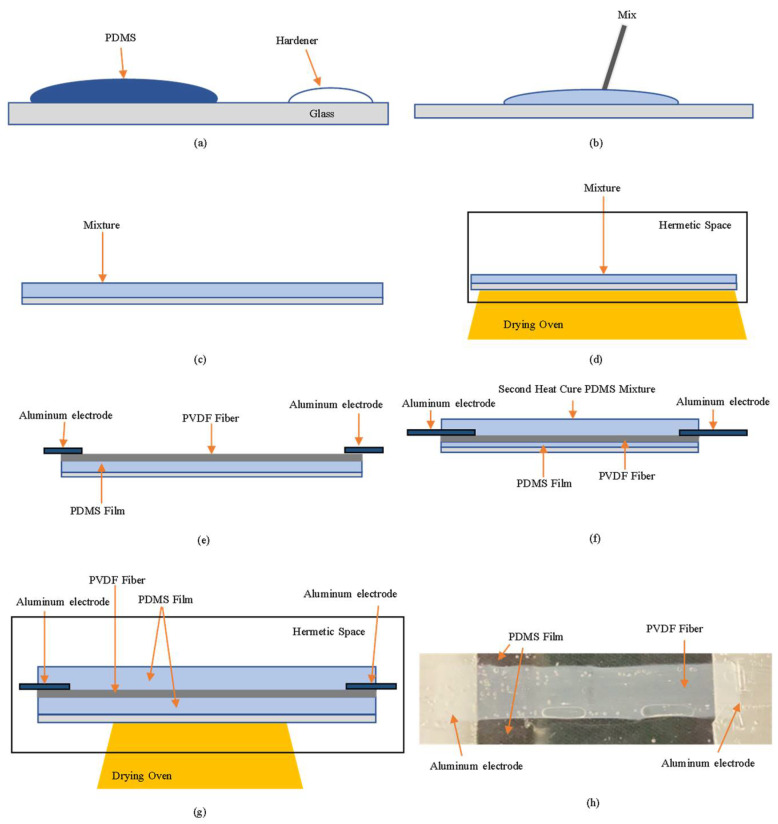
(**a**) PDMS and curing agent are formulated at a ratio of 10:1; (**b**) Thoroughly stir with a stirring bar; (**c**) Pour on the glass and wait for the bubbles to evaporate; (**d**) Put into an oven and perform closed heating in parallel. The temperature is 110 °C; (**e**) PVDF fiber film is laid out on PDMS film. Aluminum electrodes are attached to both ends of the PVDF fiber film; (**f**) Repeat steps (**a**,**b**), then pour the mixture over the element from (**e**); (**g**) Put into an oven and perform closed heating in parallel. The temperature is 40 °C; (**h**) Actual diagram of self-powered strain sensor.

The self-powered strain sensor is composed of a PDMS film and PVDF fiber film. As shown in Figure 5, the PDMS and the curing agent are mixed in a ratio of 10:1. The PDMS film is made in a heat-cured manner to produce a transparent film with elasticity. Thanks to its excellent stretchability, the heat-cured PDMS film is often used as a substrate for the microelement. The PVDF fiber film is made through the process of NFES, and the mixed solution is composed of PVDF, dimethyl sulfoxide (DMSO), and acetone (CH_3_COCH_3_). Through NFES, the PVDF mixed solution can be turned into a solid-state fiber to make a piezoelectric element. Then. The PVDF fiber film, PDMS film, and Al electrode can be laminated in sequence to form a self-powered strain sensor. If the PVDF fiber film is not closely adhered to the PDMS film and is directly exposed to air, the piezoelectric performance of the PVDF fiber film will drop very quickly and easily. Therefore, we flatten the PVDF fiber film thread onto the PDMS film, fix the two ends of the Al electrode to be thermally cured again, and package the bonded PDMS film and PVDF fiber film. This time, the thermal curing temperature drops about 40 °C and the time increases to about 3 h. Through the second thermal curing process, a complete self-powered strain sensor is made.

**Figure 6:**
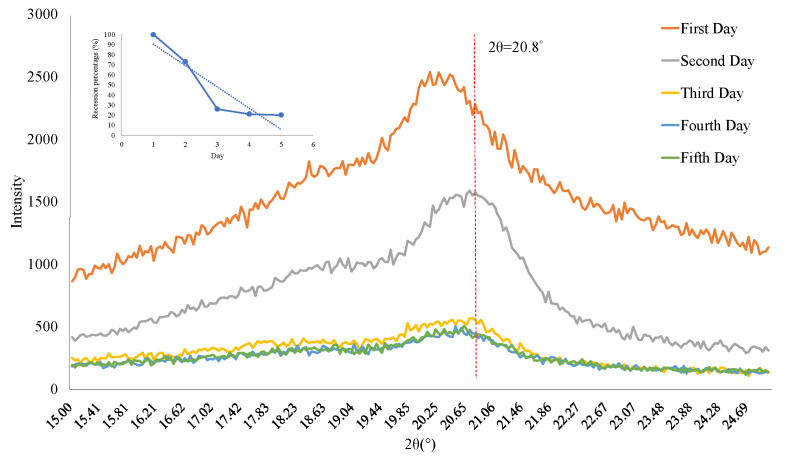
XRD pattern for self-powered strain sensors.

In this experiment, the internal crystallization of PVDF fibers measured by XRD must transform α-phase crystals without piezoelectric properties into β-phase crystals so that the PVDF fibers will have piezoelectric properties. The crystalline strength of the β phase at 20.8° and 2.5 kV has better strength than other voltages. The XRD spectrum of the self-powered strain sensor has a peak at 20.8° caused by β-crystal reflection, as shown in Figure 6, but the peak at 20.8° will fade with time, so the piezoelectric element is time-bound. Therefore, to explore the impact of time on PVDF fiber, the downward trend at the value of 20.8° for the XRD spectrum of the piezoelectric element manufactured using NFES technology can be assessed. After more time, it can be found that the peak of 20.8° exhibits a fading trend; the peak of the first day is the highest, the peak of the third day begins to fade to 20% of the first day, and the falling amplitude begins to approach saturation.

## 4. Results

### 4.1. Static Measurement

**Figure 7:**
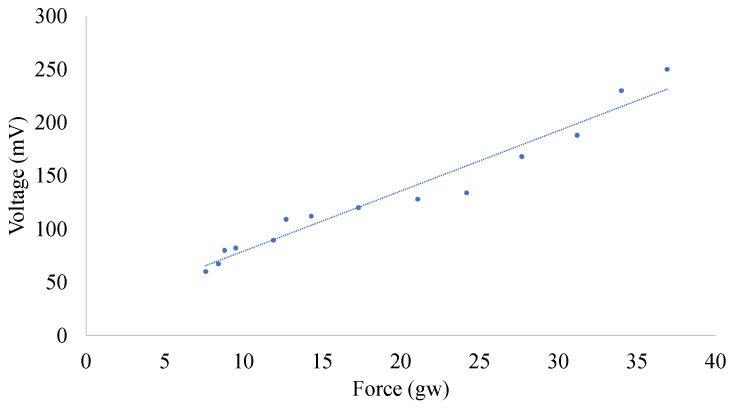
Sensitivity ratio of self-powered strain sensors for force (gw) and voltage (mV).

In this paper, the sensitivity of self-powered strain sensors was tested using a patting experiment, and PVDF fibers were made using the NFES method. To avoid experimental errors caused by a reaction force when patting, the self-powered strain sensors were lifted away from the testing platform, and we recorded the beat force at different rotational speeds. The piezoelectric effect increased as the speed increased, as shown in Figure 7. The sensitivity of the self-powered sensor was 2.371. This value was obtained after multiple measurements. Therefore, the self-powered strain sensors’ performance was extremely stable.

**Figure 8:**
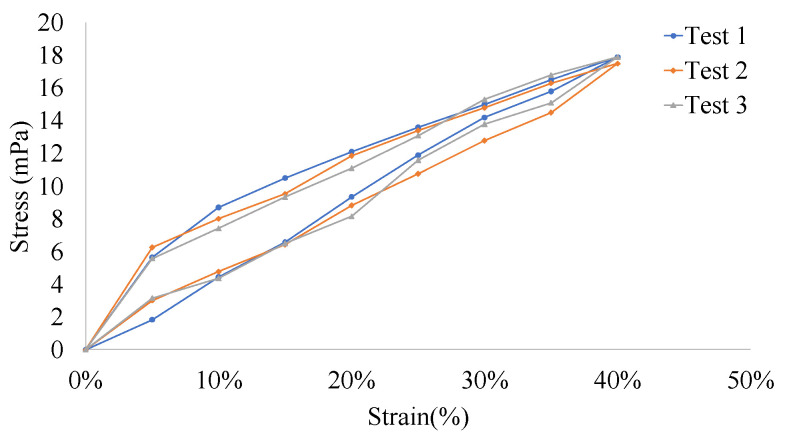
Hysteresis ring for flexible self-powered strain sensor.

Zhang et al. [22] made a wearable sensor using PDMS in a ratio of 10:1, which showed negligible hysteresis, indicating a stable mechanical performance under cyclic loading conditions, without a preprocessing cycle before stretching the PDMS. A dynamic stretching test was conducted to study the mechanical properties of self-powered strain sensors in a repetitive-stretch state, where we experimented with a full-sensor state, directly stretching the self-powered strain sensors using a stretcher to generate a hysteresis loop, as shown in Figure 8. It was found that the sensors had no notable hysteresis at 0–40%, so good stress and strain transfer could be achieved with the PVDF fiber film, and when the strain changed to 35%, the PVDF fiber film was found to demonstrate breakage with a decrease in the piezoelectric effect and sensitivity, indicating that the experimental range of strain detection is 0–35%.

### 4.2. Dynamic Measurement

**Figure 9:**
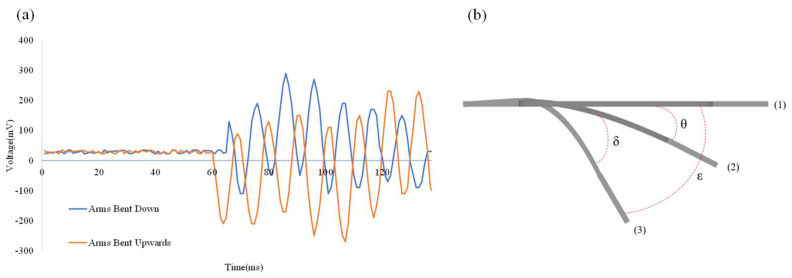
(**a**) Installation of the sensor on the robotic arm, with blue signal for arm down and orange for arm up; (**b**) bending diagram of piezoelectric strain sensor. (1) Initial position of the sensor. (2) With the sensor position of (1) as the zero plane, bending downward θ. (3) With the sensor position of (2) as the zero plane, bending downward δ, so the angular difference with (1) is ε.

The study sought to demonstrate that the self-powered strain sensor can be applied on a robotic arm. To combine the results for the sensor signal with the results for the bending test, the bending of the robotic arm was tested under conditions that demonstrated the PVDF fibers’ bending range. When the robotic arm was down, as shown in blue in Figure 9a, and when the robotic arm was up, as shown in orange in Figure 9a, the opposite waveforms were found, so it could be determined that the movement direction of the self-powered strain sensor was up or down. Figure 9b is a bending diagram for the self-powered strain sensor, with the piezoelectric output voltage $E_{1}$ shown in Figure 9b(1), bending the sensor θ to the piezoelectric output voltage $E_{2}$ shown in Figure 9b(2), and producing a piezoelectric output voltage variation $\Delta E_{1}$ from Figure 9b(1,2).

(8) $$\Delta E_{1}=E_{2}-E_{1}\;\;$$

$\Delta E_{1}$ Corresponding to Figure 9b, a bending angle value of θ may be obtained. Re-bend the sensor δ to the piezoelectric output voltage $E_{3}$ shown in Figure 9b(3), producing a piezoelectric variation $\Delta E_{2}$ from Figure 9b(2,3).

(9) $$\Delta E_{2}=E_{3}-E_{2}\;$$

$\Delta E_{2}:$ Corresponding to Figure 9b, a bending angle value of δ may be obtained. If Figure 9b(1) is the zero plane, the bending angle of Figure 9b(3) is *ε*.

(10) ε=θ+δ 

In this way, the data on the self-powered strain sensor can be analyzed and the continuous bending angle can be determined. In that context, with the above theoretical basis, a cumulative angle algorithm was designed to accumulate the angular change of the object over a unit of time or the cumulative displacement of the object over the entire period of motion. The self-powered strain sensor can improve on traditional piezoelectric sensors, which can only be sensed once. The new sensor will have the ability to continuously measure in real-time, thus enabling the use of a PVDF fiber sensing element for long-term monitoring of structural techniques.

**Figure 10:**
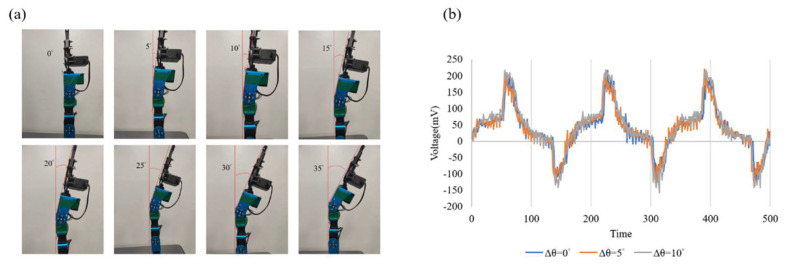
(**a**) Bending diagram of robotic arms from 0 to 35°; (**b**) Piezoelectric effects at bending angles of 0, 5, and 10°.

To verify that the self-powered strain sensor can sense the bending angle, a robotic arm was used for dynamic testing, where the self-powered strain sensor was pasted on the robotic arm to measure different angles, from 0 to 35°. As shown in Figure 10a, the self-powered strain sensor needs to be flatly attached to the robotic arm; if it cannot be flatly attached, the detected piezoelectric effect will be distorted, so tight and flat attachment is an important factor for accurate measurement. There is a piezoelectric effect of about 343 mV at 0°. There is a piezoelectric effect of about 353 mV at 5°. Then, 10° has a piezoelectric effect of about 378 mV. The piezoelectric effect varies from 0 to 5° by about 10 mV. The changes are too small to judge. The piezoelectric effect varies from 0 to 10° by about 35 mV. Therefore, the piezoelectric effect changes up to 35 mV. It can be seen from Figure 10b that the piezoelectric effect waveform from 0 to 5° is too small, so the minimum measurement start point is set to 10°. The self-powered strain sensor resolution is 5°.

**Figure 11:**
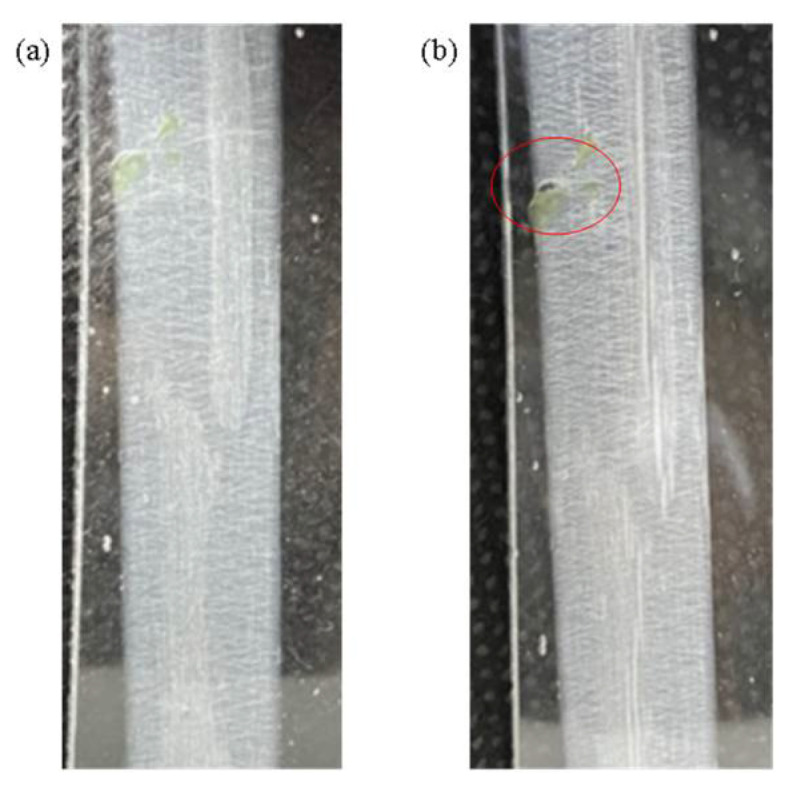
(**a**) Sensor angle at 0°; (**b**) The sensor angle at 35°; the red circle is where the PVDF fiber breaks.

The element was subjected to a bending test to measure the output voltage of the self-powered strain sensor, and the output voltage of the sensor was observed at different angular bending states. With the arm downward, it was found that as the bending angle increases, and the change of the piezoelectric effect (output voltage) also rises. From 0 to 10°, the angle change is 10°, and the output voltage is 37.6 mV; from 0 to 35°, the angle change is 35°, and the output voltage is 54.4 mV, but the angle variation is 30 to 35°, and the output voltage is not found to increase with the angle, but to decrease, because the PVDF fiber has been broken. Repeated experiments revealed the same phenomenon, as shown in Figure 10b. To achieve continuous control in different states, under the same bending conditions, different initial and terminal positions were designed to explore whether continuous control could be achieved. At different bending angles and different initial positions of bending, the output voltage was different, but under the same bending angle, the change of the output voltage was the same. When the angular variation is 10°, the piezoelectric variation of the arm bending downward in six different states is close to the piezoelectric variation of 37.6 mV from 0 to 10° at different initial and terminal positions. The same result is also obtained in other states, so it can be used to determine the bending of the strain sensor according to this characteristic. The piezoelectric variation of the arm’s upward angle is 20°, for example, from 0 to 20°, and from 15 to 35°, there is a downward trend because the PVDF fiber breaks, reducing the piezoelectric effect, as shown in Figure 11.

## 5. Application for Sensing of Bending Angle Structure

**Figure 12:**
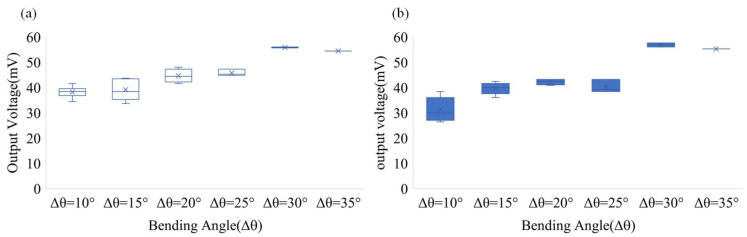
Piezoelectric variation of robotic arm at different angles in different directions. (**a**) Piezoelectric variation of robotic arm when falling at different bending angles; (**b**) Piezoelectric variation of robotic arm when rising at different angles.

In the measurement of the bending angle of the structure, the self-powered strain sensor is closely attached to the robotic arm to measure the bending at different angles. Figure 12 shows the piezoelectric variation of the robotic arm at different bending angles (Δθ): 10, 15, 20, and 35°. For example, when the bending angle Δθ = 15°, the test combination has 0~15°, 5~20°, 10~25°, …, 20~35°. As shown in Figure 12a, when the robotic arm is bent downward, the output voltages generated by each bending angle are different. When the variation angle Δθ = 10°, the output voltage of the self-powered strain sensor is 38.2 mV; when the variation angle Δθ = 15°, the output voltage is 39.12 mV; when the variation angle Δθ = 35°, the output voltage is 54.4 mV. Similarly, Figure 12b shows an approximate result for the change in the bending angle when the robotic arm is bent upward. According to the above results and combined with the angle accumulation algorithm, the angle change of each segment and the whole angle change of the measured object in a unit of time can be accumulated. For example, when the robotic arm is bent upward by 10°, the voltage output is 38.2 mV; when it is bent upward by 20°, the voltage output is 44.6 mV, and the whole bending angle is 30°. In this study, the angle of each segment of the robotic arm can be estimated from the voltage output of each segment, then the full angle is estimated through the angle accumulation algorithm. This can avoid the limitation where a piezoelectric sensor can only measure the variation up to a single time and cannot perform multiple measurements. In this study, the self-powered strain sensor had real-time continuous measurement capability and could monitor structural deformation for a long time, representing a suitable replacement for the traditional strain sensor.

## 6. Conclusions

In this study, a self-powered strain sensor was developed to evaluate the potential of sensors for structural sensing applications. In the static analysis, the piezoelectric element showed a good piezoelectric performance and the decay amplitude was small. In the dynamic analysis, the self-powered strain sensor did not have significant hysteresis. From the bending piezoelectric response, we learned that when the angle variation is 35°, the robotic arm’s downward bending output voltage is 54.4 mV, while the robotic arm’s upward bending output voltage is 55.2 mV. Through the bending angle experiment, the relationship between the angle change and output voltage was then determined. Further to this, we showed that when applying a self-powered strain sensor to robotic arm bend sensing, the robotic arm bend can be sensed to determine the position of the robotic arm. Finally, in this study, the self-powered strain sensor combined with the angle accumulation algorithm was applied to monitor the bending angle of a structure, achieve continuous measurement of the bending angle structure, and improve on traditional piezoelectric sensors, which can only be sensed once. As the key takeaway, self-powered strain sensors can continuously measure in real-time, thus supporting the use of piezoelectric sensors for long-term monitoring of structural techniques.

## Data Availability

Not applicable.
